# Reverse switching from the biosimilar SB2 to the originator infliximab in previously switched patients with inflammatory bowel diseases: results of a prospective long-term cohort study

**DOI:** 10.1177/17562848241301887

**Published:** 2024-11-30

**Authors:** Sarah Fischer, Moritz Donhauser, Sarah Cohnen, Konstantin Fietkau, Marcel Vetter, Maria Grübel-Liehr, Peter Dietrich, Timo Rath, Angelika Wilfer, Ludmilla Sologub, Sabine Krebs, Frank Dörje, Daniel Nagore, Sebastian Meyer, Markus F. Neurath, Raja Atreya

**Affiliations:** First Department of Medicine, University Hospital Erlangen, Friedrich-Alexander-Universität Erlangen-Nürnberg, Erlangen, Germany Deutsches Zentrum Immuntherapie, Erlangen, Germany; Deutsches Zentrum Immuntherapie, Erlangen, Germany; Department of Orthopaedic and Trauma Surgery, University Hospital Erlangen, Friedrich-Alexander-Universität Erlangen-Nürnberg, Erlangen, Germany; First Department of Medicine, University Hospital Erlangen, Friedrich-Alexander-Universität Erlangen-Nürnberg, Erlangen, Germany Deutsches Zentrum Immuntherapie, Erlangen, Germany; Deutsches Zentrum Immuntherapie, Erlangen, Germany; First Department of Medicine, University Hospital Erlangen, Friedrich-Alexander-Universität Erlangen-Nürnberg, Erlangen, Germany Deutsches Zentrum Immuntherapie, Erlangen, Germany; Deutsches Zentrum Immuntherapie, Erlangen, Germany; First Department of Medicine, University Hospital Erlangen, Friedrich-Alexander-Universität Erlangen-Nürnberg, Erlangen, Germany Deutsches Zentrum Immuntherapie, Erlangen, Germany; Deutsches Zentrum Immuntherapie, Erlangen, Germany; First Department of Medicine, University Hospital Erlangen, Friedrich-Alexander-Universität Erlangen-Nürnberg, Erlangen, Germany Deutsches Zentrum Immuntherapie, Erlangen, Germany; Deutsches Zentrum Immuntherapie, Erlangen, Germany; First Department of Medicine, University Hospital Erlangen, Friedrich-Alexander-Universität Erlangen-Nürnberg, Erlangen, Germany Deutsches Zentrum Immuntherapie, Erlangen, Germany; Deutsches Zentrum Immuntherapie, Erlangen, Germany; First Department of Medicine, University Hospital Erlangen, Friedrich-Alexander-Universität Erlangen-Nürnberg, Erlangen, Germany Deutsches Zentrum Immuntherapie, Erlangen, Germany; Deutsches Zentrum Immuntherapie, Erlangen, Germany; First Department of Medicine, University Hospital Erlangen, Friedrich-Alexander-Universität Erlangen-Nürnberg, Erlangen, Germany Deutsches Zentrum Immuntherapie, Erlangen, Germany; Deutsches Zentrum Immuntherapie, Erlangen, Germany; First Department of Medicine, University Hospital Erlangen, Friedrich-Alexander-Universität Erlangen-Nürnberg, Erlangen, Germany Deutsches Zentrum Immuntherapie, Erlangen, Germany; Deutsches Zentrum Immuntherapie, Erlangen, Germany; Pharmacy Department, University Hospital Erlangen, Friedrich-Alexander-Universität Erlangen-Nürnberg, Erlangen, Germany; Pharmacy Department, University Hospital Erlangen, Friedrich-Alexander-Universität Erlangen-Nürnberg, Erlangen, Germany; Progenika Biopharma, Derio, Spain; Department of Medical Informatics, Biometry and Epidemiology, Friedrich-Alexander-Universität Erlangen-Nürnberg, Erlangen, Germany; First Department of Medicine, University Hospital Erlangen, Friedrich-Alexander-Universität Erlangen-Nürnberg, Erlangen, Germany; Deutsches Zentrum Immuntherapie, Erlangen, Germany; First Department of Medicine, University Hospital Erlangen, Friedrich-Alexander-Universität Erlangen-Nürnberg, Ulmenweg 18, Erlangen 91054, Germany; Deutsches Zentrum Immuntherapie, Erlangen, Germany

**Keywords:** biological, biosimilar, IBD, infliximab, multiple switches, reverse switch, SB2, switch

## Abstract

**Background:**

Data regarding multiple switches including reverse switching between infliximab and its biosimilars are scarce in the field of inflammatory bowel diseases (IBD).

**Objectives:**

We investigated the clinical effectiveness as primary outcome measure after repeated switches. Secondary endpoints included C-reactive protein (CRP) levels, immunogenicity (trough levels (TL); anti-drug antibodies (ADA), safety and drug persistence.

**Design:**

This study is a prospective, single-centre, observational cohort study. IBD patients receiving originator infliximab were switched to biosimilar SB2 and then reverse switched after 96 weeks and followed up for another 48 weeks.

**Methods:**

Clinical disease activity (Harvey–Bradshaw-index (HBI) in Crohn’s disease (CD), partial Mayo score (pMS) in ulcerative colitis (UC)), CRP, TL, ADA, therapy-discontinuations and (serious) adverse events ((S)AE)) were monitored throughout the study.

**Results:**

Ninety-five patients (60 CD, 38 female) were enrolled. The median HBI was 2 (interquartile range (IQR) 1–4) at baseline and 2 (1–4) at week 48, resulting in a mean intra-individual change of 0.0 (standard deviation (SD) 1.5). The median pMS was 1 (IQR 0–1) at baseline and 0.5 (0–1) at week 48 resulting in a mean intra-individual change of 0.0 (SD 0.8). Clinical remission was achieved in 80% at baseline and 82% at week 48. Median CRP 2.0 mg/l (IQR 1.0–4.1) at baseline and 2.4 mg/l (1.1–5.2) at week 48 resulted in a mean change of 1.7 (SD 5.8) and no significant differences in CD (*p* = 0.3) and UC (*p* = 0.9). Median TL were 7.2 µg/ml (IQR 3.8–19.3) at baseline and 5.5 µg/ml (3.5–13.1) at week 48, resulting in a mean change of −1.0 (SD 7.4) with no statistical significance (CD *p* = 0.26, UC *p* = 0.41). De-novo-ADA were developed by 3.4%. The discontinuation rate was 14.7%. Safety signals were consistent with previous studies.

**Conclusion:**

Reverse switching had no impact on efficacy of infliximab therapy in our cohort of IBD patients. The switch didn’t influence immunogenicity or safety of therapy.

## Plain language summary

A clinical study investigating the interchangeability between infliximab, a previously patented biotechnological produced drug (biological) and its successor product biosimilar SB2, which is highly similar to the existing reference product, in the treatment of patients with inflammatory bowel diseases

Why was the study done? Biological drugs (like infliximab) are produced using various methods under the involvement of biotechnology. In contrary to generic drugs, due to their complex structure, biosimilars are not exactly the same molecule as the original substance. After the introduction of biosimilars, which are mostly less expensive than the original product, concerns were raised regarding the interchangeability of these substances. This study was conducted to investigate potential changes in efficacy and safety after multiple, so called switches (from the original product to its biosimilar and back). What did the researchers do? The research team switched a group of 95 patients diagnosed with inflammatory bowel disease and therefore treated with the original biologic drug infliximab to the biosimilar SB2, reverse switched them back to the original drug after 96 weeks and observed the patients for another 48 weeks. During the observational phase the disease activity, drug levels and potential side effects were monitored. What did the researchers find? The initial and the reverse switch had no relevant impact on the course of disease and additionally no safety issues occurred. During the 48 weeks observational period, 14.7% of patients discontinued therapy, which was not out of the expected range. Most potential side effects were not considered to be therapy-associated and only 4 of 95 patients discontinued treatment due to safety concerns. The drug levels remained stable during the switches. What do the findings mean? In this study, the research team found no impact of multiple switches on the disease activity in inflammatory bowel disease patients and moreover no safety issues. These findings support the practice of switching between the biologic originator substance infliximab and its biosimilars.

## Introduction

Inflammatory bowel diseases (IBD) are chronic, debilitating, immune-mediated disorders of the gastrointestinal tract, whose phenotypic entities mainly comprise Crohn’s disease (CD) and ulcerative colitis (UC). Both diseases are characterized by a relapsing-remitting course with heterogeneous phenotypes, disease courses and complications. Optimized anti-inflammatory therapy is indispensable in affected patients, as ongoing inflammation is associated with impaired quality of life, progressive bowel damage, heightened risk of complications and increased incidence of colitis-associated neoplasia.^1,2^ The advent of targeted therapies has substantially improved the therapeutic outcomes of treated patients and has become an integral part of existing therapeutic algorithms.^3^ The anti-tumour necrosis factor (TNF) antibody infliximab was the first biological therapy approved for the treatment of IBD in 1998.^4^ However, biological medications for IBD account for a significant burden of cost to healthcare systems among patients with IBD.^5,6^ The high cost of anti-TNF agents and the expiry of patents led to the development of biosimilar products with the potential to reduce the cost of treatment through competitive market practices and by addressing access inequities.^7^ In 2013, the European Medicines Agency approved the first biosimilar monoclonal antibody drug, CT-P13, for the treatment of several immune-mediated inflammatory diseases, including IBD.^8^ A biosimilar, defined as a biological medicinal product that contains a version of the active substance of an already authorized biological medicinal product (originator), must be as safe, pure, potent and efficacious as the reference product based on comprehensive comparability exercises, such that there are no clinically meaningful differences (therapeutic equivalence).^9^ The European Crohn’s and Colitis Organization (ECCO) has stated that prescribing biosimilars and switching from originators to biosimilars in patients with IBD are acceptable, provided that patients are well informed and adequately monitored.^10^ Although the approval for biosimilar use in IBD has been based on extrapolation of rheumatologic studies, a growing body of literature supports their use in IBD and the efficacy and safety of unidirectional switching from the reference product. These studies include the double-blind NOR-SWITCH study, which reported that the incidence of disease worsening in switched patients was within the predefined absolute margin set for non-inferiority of 15% to the incidence in patients who remained on the originator.^11^ In explorative subgroup analyses, efficacy, safety and immunogenicity of both the originator and biosimilar infliximab were comparable in CD and UC in the NOR-SWITCH main and extension trials.^12^

Other double-blinded randomized controlled trials involving anti-TNF agents also demonstrated non-inferiority between remaining on an originator and switching to a biosimilar.^13^ Data accumulated from real-life cohorts on clinical efficacy, safety and immunogenicity of biosimilars likewise show comparable outcomes with the originator infliximab in both, anti-TNF-naïve and switched patients.^14–18^ Switching from originator infliximab on a biosimilar due to economic reasons is now part of routine care in the treatment of IBD. Data regarding multiple switches between the originator infliximab and its biosimilars on the contrary are scarce and the question of whether biosimilars could be treated analogous to generic drugs remains to be answered.

Physicians must also acknowledge the process of reverse switching, which covers all returns to previously used infliximab, regardless if originator or biosimilar, in their therapeutic approaches in the current IBD treatment landscape.^19^ Multiple switching is the term routinely used to describe at least three therapy transitions, or alternating between the reference product and its biosimilar product as well as between biosimilars. Based on a definition of this nature, multiple switching may not be mutually exclusive from reverse switching.^20^

There is currently scarce evidence regarding reverse switching in patients with IBD. Reverse switching to the originator anti-TNF after an initial switch from originator to a biosimilar anti-TNF is often driven by perceived loss of response or adverse effects. This process of reverse switching is frequently attributed to a nocebo effect, defined as a biochemically or pharmacologically unexplainable unfavourable outcome, after a switch from originator to biosimilar anti-TNF.^21,22^ Currently, reverse switching (also called switchback^23^) due to loss of response is not recommended because of a lack of clinical and scientific evidence.^10^ Furthermore, reverse switching can be caused due to an economic-driven change of tender arrangements that leads to a non-medical change from an anti-TNF biosimilar to the originator in patients with IBD.^21^ Altogether, data on reverse and multiple switching still need to be extended and corroborated to elucidate its practical consequences.

In 2017, we initiated a cohort study investigating 144 IBD patients under originator-infliximab therapy and prospectively observed the cohort for an 80-week period after switching to the biosimilar SB2. The infliximab biosimilar SB2 (Flixabi^®^, Renflexis^®^), was approved by the European Medicines Agency in 2016 and by the Food and Drug Administration in 2017 for the treatment of patients with IBD. The switch did not impact the primary outcome measure clinical disease activity at week 80, reflected by a mean change of disease activity scores of −0.4 (standard deviation (SD) 2.0) in CD and 0.1 (SD 1.3) in UC. The median TLs remained stable and median C-reactive protein (CRP) levels were within normal ranges throughout the study. We concluded a switch from originator infliximab to the biosimilar SB2 to be safe and have no substantial impact on the clinical effectiveness and immunogenicity in the treatment of IBD patients. However, the topic of reverse switching is of additional heightened interest, as this might represent an occurring scenario in clinical practice. It needs to be studied if patients might have a higher risk for loss of response if re-exposed to a previously paused medication due to higher risk of sensitization when using exactly the same drug as before the first switch. In the current study, we followed up our cohort of initially switched patients from originator infliximab to biosimilar SB2 for another 16 weeks. All of these patients were then reverse switched from SB2 to originator infliximab and followed up for another 48 weeks to assess the effectiveness, immunogenicity and safety of our prospectively followed up IBD patient cohort.

## Materials and methods

### Study design

The trial was designed as a single-centre, prospective, longitudinal, observational cohort study at the outpatient Clinic for IBD of the Medical Department 1 of the University Hospital Erlangen, Germany. All patients received careful counselling from the treating physician before the initial and the reverse switch. Consent for the switch was obtained by the treating physician. Infliximab was used according to the recommended indications, dosing and infusion intervals. Change of dosing, infusion interval and discontinuation of treatment were permitted and subjected to the treating physician’s choice. The application method was not changed during the trial. All patients received infliximab intravenously at all times. Blood sampling for measurement of trough levels (TL), anti-drug-antibodies (ADA) and CRP took place before every infliximab administration. The use of potentially immunosuppressant co-medication such as steroids, thiopurines or methotrexate was registered. All adverse events (AEs) were registered. All data were captured as part of routine clinical practice. As a non-interventional study, outcomes were measured according to the usual patient visit schedule, with flexibility around timeline milestones. The sample collection was previously approved by the ethical committee and the institutional review board of the Friedrich-Alexander-University of Erlangen-Nürnberg (proposition approval number: 40_16B). The reporting of this study is in compliance with the Strengthening the Reporting of Observational Studies in Epidemiology (STROBE) statement (Supplemental Material).

### Patients

All patients with IBD receiving originator-infliximab therapy were consecutively switched between February and April 2017 to the biosimilar SB2 for non-medical reasons and followed up till January and April 2019, when they were all consecutively reverse switched on originator infliximab. Afterwards, the entire cohort of patients was followed up for an additional 12 months. All patients were at least 18 years old with a confirmed diagnosis of IBD according to the ECCO criteria, including CD or UC. Patients with infliximab treatment for chronic pouchitis following proctocolectomy and patients with ostomies were excluded from this study. Baseline characteristics included sex, age, disease entity, Montreal classification, onset of disease, duration of previous infliximab treatment before the index switch, body-mass-index, infliximab dosing and interval as well as exposure to potentially immunosuppressive co-medication were recorded.

### Outcome measures

The primary outcome measure was clinical disease activity at weeks 24 and 48 after the reverse switch from biosimilar SB2 to originator infliximab (baseline). Secondary outcome measures include the CRP levels, TL, the development of ADA and safety. All parameters were assessed by the treating physician at every patient visit and analysed at the weeks −96, −48, −24, 0 (baseline, the second switch) and 24, 48.

#### Clinical disease activity

Clinical disease activity was evaluated using the Harvey–Bradshaw index (HBI) in CD patients and the partial Mayo score (pMS) in UC. Patients with a HBI of 0–4 or a pMS of 0–1 were defined to be in clinical remission. Mild clinical disease activity was defined as an HBI of 5–7 or a pMS of 2–4, moderate disease activity as an HBI of 8–16 or a pMS of 5–7 and severe disease activity was assumed for any higher values.^24,25^

#### C-reactive protein

The CRP was measured at every patient visit. Levels below 5.0 mg/l were considered to be negative.

#### TLs and anti-drug-antibodies

Blood sampling for the measurement of infliximab TL and ADA took place prior to every administration using Promonitor^®^ tests of Progenika Biopharma (Derio, Spain), an enzyme-linked immunosorbent assay validated for the measurement of either the originator infliximab or any approved biosimilar as well as the corresponding ADA. The serum probes were diluted to 1:500, resulting in a measurable TL range of 0.2–32.0 μg/ml. Probes with higher TL were diluted by 1:3000 to achieve evaluable results. TL above 35 µg/ml were assumed to be a sampling error and therefore excluded. TLs between 3 and 7 µg/ml were considered to be within the optimal therapeutic range.^26^ Any measurable ADA level was accounted as ADA-positivity. The results of TL and ADA measurements were not shared with the treating physician and did therefore not influence the treatment scheme. All changes in dosing according to the recommended indications and dosages were permitted at the physician’s discretion based on clinical disease activity or endoscopic picture.

#### Drug persistence

Discontinuation and the underlying reason, if available, were registered. All potentially therapy-associated treatment discontinuations and all patients lost to follow-up were considered to be informative drop-outs. For patients who paused infliximab treatment and were re-introduced during the study, all parameters (including clinical disease activity, TL, ADA and CRP) were excluded until induction therapy was completed.

#### Safety

Every occurring AE was registered. Every potentially life-threatening, permanently disabling or events leading to hospitalization were listed as severe adverse events (SAEs). All AEs were categorized for their relation to the treatment as ‘not related’, ‘possibly related’, ‘probably related’ and ‘certainly related’ by the study team.

### Statistics

Prior to the initiation of the study, we did not perform a sample size calculation due to the predetermined sample size, as all patients in our outpatient Clinic for IBDs with an initial switch from originator infliximab to the biosimilar SB2 and still on SB2 treatment 2 years later were included in the present study. Clinical disease activity scores, CRP levels, TL and ADA were evaluated at 8-weekly main visits. If multiple visits for the same patient fell in the 8-week window, the visit closest to the regular 8-week interval was used for the analysis. AEs, SAEs and treatment discontinuation were registered at the time point of occurrence. Categorical variables (clinical disease severity, TL in therapeutic range, ADA, AE, SAE, treatment discontinuations) were described using counts and proportions, while metric variables (HBI, pMS, CRP, TL) were described using the median (interquartile range, IQR). Change scores compared to baseline were summarized using the mean (SD).

Metric variables were visualized using a combination of box- and bee swarm plots that include the individual measurements. Drug persistence was analysed using the Kaplan–Meier estimator for the time to originator-infliximab discontinuation, where censoring included loss to follow-up, end of study and therapy interruptions. The parameters TL, CRP level and 8-week-dosing were analysed for mean differences 1 year before and after the second switch, using mixed regression models with a phase effect, separately for each IBD cohort. The infliximab dosing was normalized to an 8-week interval and scaled by body weight, to generate comparable values. The analysis was limited to patients, who were still under infliximab therapy 1 year after the second switch, using available data from all visits. The parameters were adjusted for the baseline characteristics body mass index, duration of therapy, age and sex, as well as the dosing preceding each visit (a potential confounder for TL). Outcomes were transformed to satisfy distributional assumptions: TL was modelled on the square-root scale, CRP and 8-week-dosing were modelled logarithmically. Based on the fitted models, adjusted means for the SB2-infliximab- and the originator-infliximab-phase (each with a 95% confidence interval, 95% CI) were calculated and tested for a phase effect. All statistical analyses were carried out in the statistical software environment R 4.1.3 (R Core Team, 2022).

## Results

Over the complete observation period, 282 patient years were taken into account including 94 patient years from the reverse switch onwards. A total of 2276 patient visits were registered. In 80% of the observations, all clinical scores, TL, ADA and CRP were measured and in an additional 19% of the visits, at least clinical scores were determined. In only 1% of the cases, no data were available.

### Patient characteristics

We included 95 patients (63% CD, 40% female) and evaluated them for a median of 158 weeks (range 105–171), including a median 56 weeks (range 0–65) after the reverse switch. At the time of the first switch, patients were aged a median of 39 years (range 19–69) with a median disease duration of 8 years (range 1–48) and a previous infliximab therapy of 33 months (range 1–103). The main proportion of patients had extended disease (Montreal classification 62% L3 in CD; 60% E3 in UC). All patient characteristics are listed in Table 1. No patient requested to switch back to SB2 after the reverse switch during the observation period.

**Table 1.** All patient characteristics at time of the first switch are listed in this table.

Disease entity (*n* (%))		
CD	60 (63%)	
UC	35 (37%)	
Montreal classification	CD	UC
*n* (%)	A1: 7 (12%)	E1: 0 (0%)
	A2: 45 (75%)	E2: 14 (40%)
	A3: 8 (13%)	E3: 21 (60%)
	L1: 13 (22%)	
	L2: 10 (17%)	
	L3: 37 (62%)	
	L4: 13 (22%)	
	B1: 16 (27%)	
	B2: 10 (17%)	
	B3: 34 (57%)	
	*p*: 18 (30%)	
Gender		
Female	38 (40%)	
Male	57 (60%)	
Age (years)		
Median (range)	39 (19–69)	
Disease duration (years)		
Median (range)	8 (1–48)	
Body mass-index (kg bw/m^2^)		
Median (range)	25.2 (16.5–50.1)	
Duration of previous infliximab treatment (months)		
Median (range)	33 (1–103)	
Clinical disease activity (*n* (%))		
Remission	70 (74%)	
Mild	18 (19%)	
Moderate	7 (7%)	
Severe	0 (0%)	

bw, bodyweight; CD, Crohn’s disease; UC, ulcerative colitis.

### Clinical disease activity

**Figure 1.**
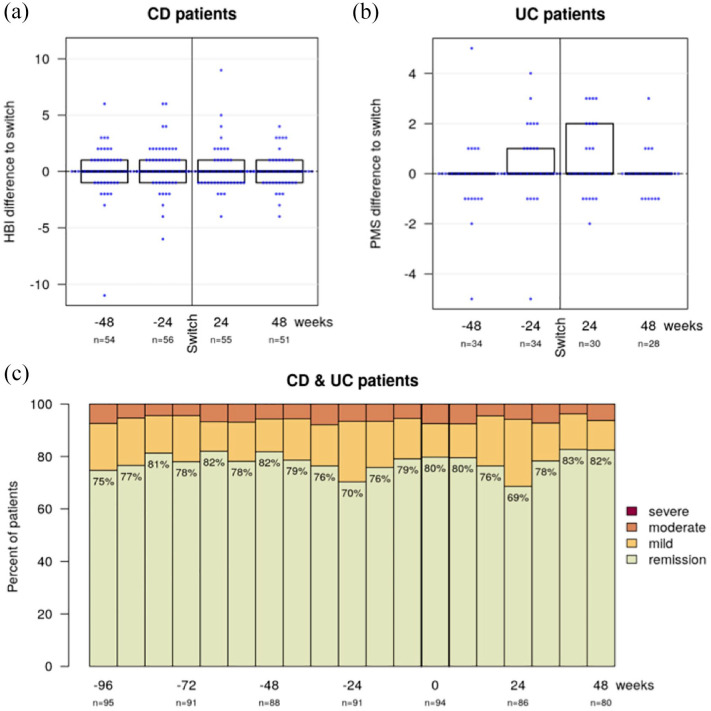
These combined bee swarm and boxplots illustrate the change of disease activity scores compared to baseline for 48 weeks before and after the second switch in Crohn’s disease (a) and ulcerative colitis (b) patients. The proportion of patients in remission, with mild or moderate disease activity (c), remained stable during the observation period. There were no cases of severe disease activity.

For CD patients the median HBI was 2 (IQR 1–4) at week −96, 2 (1–4) at week −48, 2 (1–6) at week −24, 2 (1–4) at baseline (week 0), 2 (1–5) at week 24 and 2 (1–4) at week 48, with a mean intra-individual change of the HBI compared to baseline of 0.1 (SD 2.2) at week −48, 0.4 (2.0) at week −24, 0.2 (1.9) at week 24 and 0.0 (1.5) at week 48 (Figure 1(a)). For UC patients the median pMS was 1 (0–2) at week −96, 0 (0–1) at week −48, 1 (0–2) at week −24, 1 (0–1) at baseline (week 0), 1 (0–2) at week 24 and 0.5 (0–1) at week 48, with a mean intra-individual change of the pMS compared to baseline of the pMS of −0.1 (SD 1.4) at week −48, 0.3 (1.5) at week −24, 0.5 (1.4) at week 24 and 0.0 (0.8) at week 48 (Figure 1(b)).

Among all IBD patients, clinical remission was achieved in 75% at week −96, 82% at week −48, 70% at week −24, 80% at baseline (week 0), 69% at week 24 and 82% at week 48. The proportions of mild disease activity in these weeks were 18%, 12%, 23%, 13%, 26% and 11%, respectively, and for moderate disease activity 7%, 6%, 7%, 7%, 6% and 6%, respectively (Figure 1(c)). No patients experienced severe disease activity. During the follow-up period, no clinically relevant changes in the disease activity were observed.

### C-reactive protein

**Figure 2.**
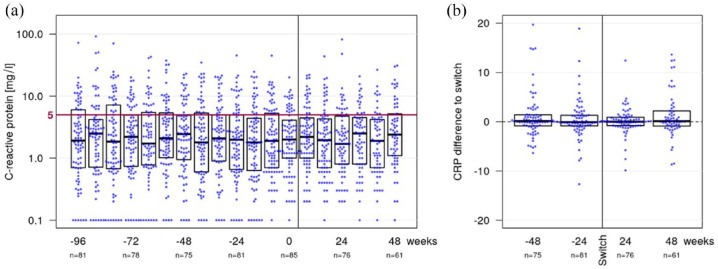
The combined bee swarm and boxplot (a) shows CRP levels in all IBD patients during the observation period. The red line indicates the threshold value of 5 mg/l. All measured values ⩽5 mg/l are considered negative. The combined bee swarm and boxplot (b) shows the change in CRP compared to baseline for 48 weeks before and after the second switch. CRP, C-reactive protein; IBD, inflammatory bowel disease.

Among all IBD patients, the CRP level had a median of 1.9 mg/l (IQR 0.7–6.0) at week −96, 2.5 mg/l (0.9–4.9) at week −48, 2.0 mg/l (0.7–4.9) at week −24, 2.0 mg/l (1.0–4.1) at baseline (week 0), 1.7 mg/l (0.8–4.8) at week 24 and 2.4 mg/l (1.1–5.2) at week 48 (Figure 2(a)). The corresponding mean intra-individual change of the CRP level compared to baseline were 1.0 (SD 4.6) at week −48, 0.9 (6.3) at week −24, 1.4 (9.2) at week 24 and 1.7 (5.8) at week 48 (Figure 2(b)).

The regression analysis revealed no phase effects before versus after the second switch. In CD patients, the adjusted mean in CRP was 1.9 mg/l (95% CI 1.4–2.6) during the SB-infliximab phase and 2.1 mg/l (1.6–2.8) during the originator-infliximab-phase resulting in no statistical phase effect (*p* = 0.30). In UC patients the adjusted mean in CRP was 2.2 mg/l (95% CI 1.3–3.8) during the SB2-infliximab phase and 2.3 mg/l (1.4–3.8) during the originator-infliximab phase resulting in not statistically significant differences before and after the second switch (*p* = 0.97) either. After the reverse switch, no clinically relevant changes in the CRP levels were observed.

### Pharmacokinetics

#### Trough levels

**Figure 3.**
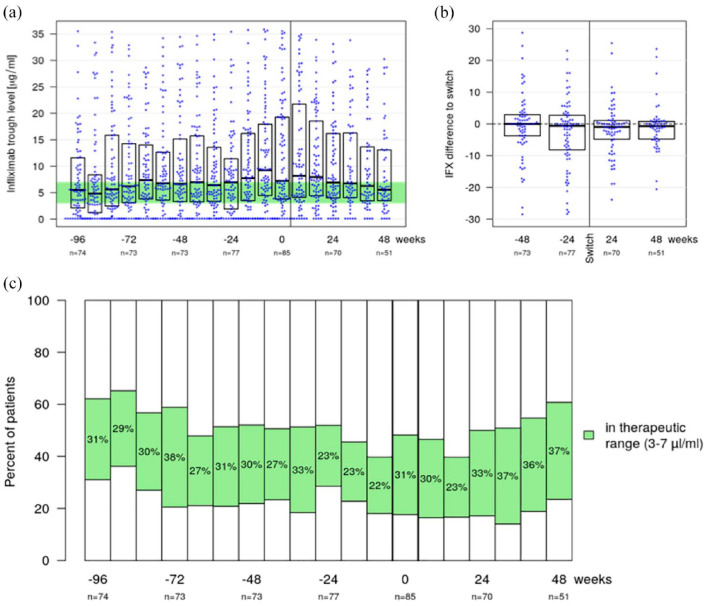
Combined bee swarm and boxplot (a) displays TL for all IBD patients over the complete observation period. The optimal TL range was considered between 3 and 7 µg/ml and is highlighted (green). Combined bee swarm and boxplot (b) illustrates the change of TL compared to baseline for 48 weeks before and after the second switch. Stacked bar chart (c) shows the proportion of patients within the considered optimal TL range, where the white bars represent the proportion of patients with higher and lower TLs. IBD, inflammatory bowel disease; TL, trough level.

Among all IBD patients the infliximab TL had a median of 5.5 µg/ml (IQR 2.1–11.6) at week −96, 6.7 µg/ml (3.3–15.1) at week −48, 7.0 µg/ml (1.9–11.4) at week −24, 7.2 µg/ml (3.8–19.3) at baseline (week 0), 6.9 µg/ml (4.0–16.2) at week 24 and 5.5 µg/ml (3.5–13.1) at week 48 (Figure 3(a)). The corresponding mean intra-individual change in TL compared to baseline was −0.4 (SD 9.8) at week −48, −2.8 (10.6) at week −24, −1.7 (8.1) at week 24 and −1.0 (7.4) at week 48 (Figure 3(b)).

Considering solely patients who completed treatment for at least 48 weeks after the second switch TLs were a median of 5.0 µg/ml (2.3–7.5) at week −96, 5.9 µg/ml (3.3–11.2) at week −48, 5.5 µg/ml (1.7–9.7) at week −24, 6.5 µg/ml (3.8–14.8) at baseline (week 0), 5.1 µg/ml (3.7–10.5) at week 24 and 5.5 µg/ml (3.5–13.1) at week 48.

TL within the therapeutic range were detected in 31% of all IBD patients at week −96, 30% at week −48, 23% at week −24, 31% at baseline (week 0), 33% at week 24 and 37% at week 48. The corresponding proportions of patients above the therapeutic range were 38%, 48%, 48%, 52%, 50% and 39%, respectively, and 31%, 22%, 29%, 18%, 17% and 24% were below the therapeutic range at the respective time point (Figure 3(c)).

Regression analysis showed no phase effects in TL. During the SB2-infliximab phase the adjusted mean TL was 8.9 µg/ml (95% CI 7.2–10.8) in CD patients and 9.1 µg/ml (5.9–13.0) in UC patients. During the originator-infliximab-phase the adjusted mean TL was 9.6 µg/ml (7.6–11.8) in CD patients and 9.6 µg/ml (6.2–13.8) in UC patients, resulting in not statistically significant phase effects (*p* = 0.26 in CD; *p* = 0.41 in UC). There were no clinically meaningful changes in the TL.

#### Anti-drug antibodies

**Figure 4.**
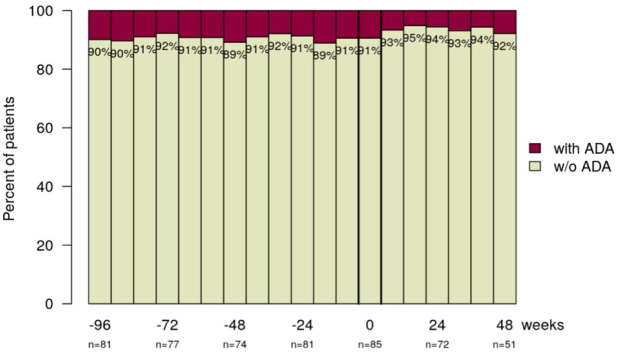
The majority of patients have not developed ADA during the observational period, indicated by this stacked bar chart. ADA, anti-drug antibodies.

ADAs were detected in 10% of the IBD patients at week −96, 11% at week −48, 9% at week −24, 9% at baseline (week 0), 6% at week 24 and 8% at week 48 (Figure 4). After the reverse switch (baseline) three patients (3.4%) developed new ADA and two (2.3%) patients lost ADA-positivity. The majority of patients (87.3%) did not develop ADA at any time point.

#### Dosing of infliximab

The dosing of infliximab at week 48 compared to baseline was stable for 40% of the patients, while 39% received lower and 21% a higher dosing. For 67% of the IBD patients, the treatment schedule remained unchanged at week 48, while 11% received infliximab in a shorter and 22% in a prolonged interval.

The regression analysis revealed no phase effects in 8-week dosing before versus after the second switch. For CD patients, the adjusted mean in the 8-week-dosing was 10.2 mg/kg body weight (bw) (95% CI 9.0–11.6) during the SB2-infliximab phase and 10.3 mg/kg bw (9.0–11.8) during the originator-infliximab-phase, resulting in a non-significant change (*p* = 0.87). For UC patients the adjusted mean in the 8-week-dosing was 8.1 mg/kg bw (6.7–9.8) during the SB2-infliximab phase and 8.3 mg/kg bw (6.9–10.0) during the originator-infliximab-phase, resulting in no significant changes (*p* = 0.54). There was no evidence for a general need for dose intensification after the second switch.

#### Immunosuppressive co-medication

A total of 19 (20.0%) patients received immunosuppressive co-medication at any time point during the observation period. Co-medication was necessary for the treatment of IBD in 11 (11.6%) patients, while 8 (8.4%) patients received it for the treatment of rheumatologic, dermatologic or otologic disorders.

Immunosuppressive co-medication was composed of 6-mercaptopurine (1), azathioprine (3), methotrexate (7) and an antibody against the interleukin 36-receptor (1). Short-term prednisolone therapy was necessary in 14 (14.7%) patients, of whom 7 received it after the reverse switch.

At baseline (week 0) 7 (7.4%) patients received immunosuppressive co-medication (one azathioprine, one 6-mercaptopurine, three methotrexate, two prednisolone). After the reverse switch, additional 6 (6.3%) patients were introduced to immunosuppressive co-medication (three short-term prednisolone therapy followed by methotrexate, three short-term prednisolone therapy). One patient receiving prednisolone at baseline was introduced to azathioprine later on, the patient on 6-mercaptopurine was introduced to azathioprine and one patient received prednisolone after azathioprine therapy. At the last registered patient visit co-medication was registered in only 6 (6.3%) patients (two methotrexate, one methotrexate + prednisolone, two prednisolone mono-therapy, one azathioprine). Premedication for primary or secondary prophylaxis of infusion-related reactions was administered in 18 (18.9%) patients (6 prednisolone, 12 prednisolone plus clemastine). Premedication was permanently (from the time of onset) necessary in 5 patients but only transient in 13 patients. In only three patients premedication was initiated after the second switch. There was no signal for general need for immunosuppressive co- or premedication in this IBD cohort.

### Drug persistence

**Figure 5.**
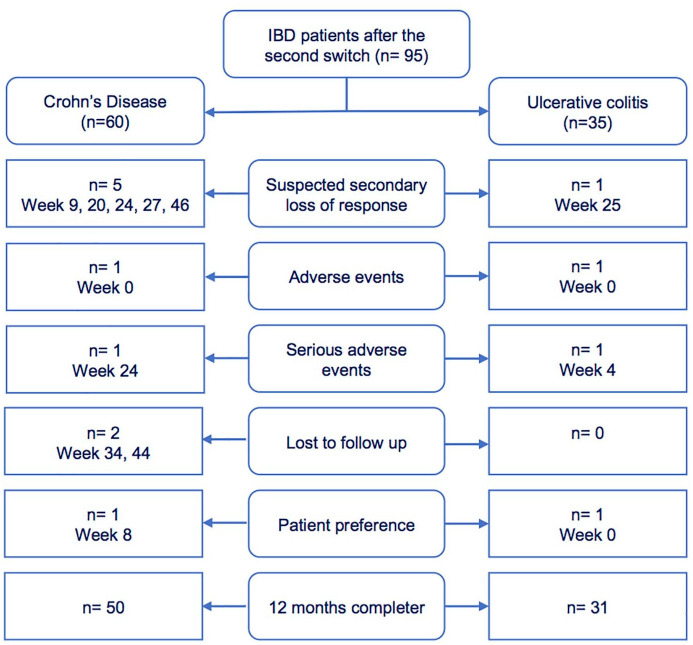
The flowchart indicates the underlying reason for every treatment discontinuation after the second switch distinguished by disease entity.

**Figure 6.**
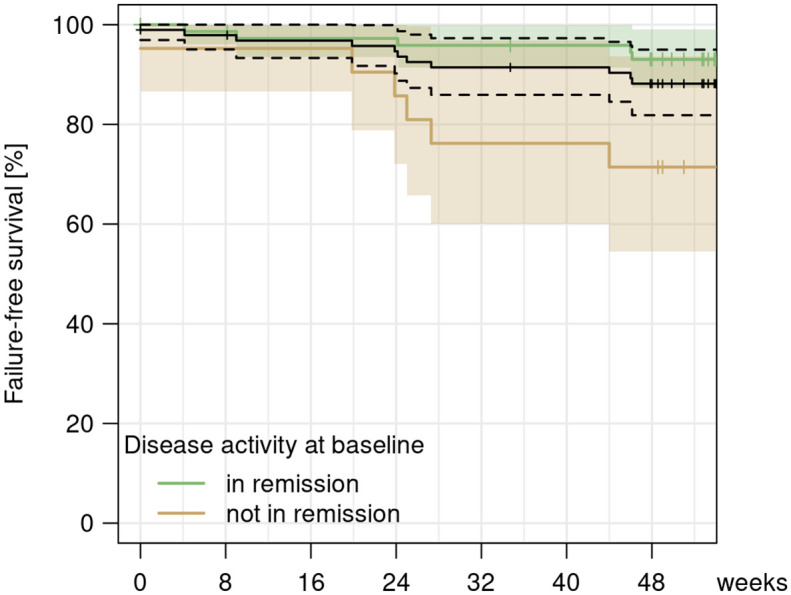
Kaplan–Meier curve showing the survival on treatment for informative criteria (secondary loss of response, adverse/serious adverse events and lost to follow-up) after the second switch for all patients (black) and differentiated for patients in remission (green) versus not in remission (brown) at the time of the switching procedure from biosimilar to originator infliximab.

Discontinuation of infliximab treatment was registered in 14 (14.7%) patients, of which 11 (11.6%) were registered as therapy failure (two AE, two SAE, six suspected secondary loss of response (SLR), one lost to follow-up) and three cases of non-informative drop-outs (one change of physician, two on patient request) (Figure 5). The AEs leading to discontinuation contained relapsing infusion-related reactions for two patients. The SAEs leading to discontinuation contained one malignancy (hepatic metastasis of a previously diagnosed neuroendocrine tumour) and one sepsis. The probability of failure-free survival was estimated as 95% (95% CI 0.90–0.99) at week 24 and 88% (95% CI 0.82–0.95) at week 48 (Figure 6). Survival on therapy was significantly higher (*p* = 0.005) in patients with clinical remission (94% at week 24, 95% CI 0.94–1.00 and 93% at week 48, 95% CI 0.87–0.99 vs. 86% at week 24, 95% CI 0.72–1.00 and 71% at week 48, 95% CI 0.55–0.94 in non-remitters) (Figure 6).

Additional 9 (9.5%) patients paused infliximab treatment between the first and the second switch (three suspected SLR, two infectious complications, one hemicolectomy due to malignancy, three patients’ preference).

Patients who discontinued the infliximab treatment were ADA positive in 30% of the cases and 70% showed sub-therapeutic TLs. There was no signal for unsuspected treatment discontinuations in this IBD cohort.

### Safety

During the follow-up period, 66 AEs were registered in 46 patients (48.4%), of which 7 were defined as serious. Serious AEs included one metastasized malignancy, one bowel resection due to an inflammatory stenosis, one hospitalization due to an acute flare of CD and one due to diarrhoea-associated hypopotassaemia, one sepsis, one surgery for scoliosis and one traumatic subarachnoid haemorrhage. AEs included arthralgia (15) and various rheumatologic, gastroenterological, infectiological, ophthalmologic and dermatologic diseases, including anaphylactic infusion reactions (3). All AEs and SAEs are listed in Table 2.

**Table 2.** All serious and non-serious adverse events are provided in this table, additionally indicating time of manifestation, disease entity of the affected patient, necessity of treatment discontinuation due to the event and correlation to the drug due to the study team’s discretion.

Patient no.	Serious adverse event	Point of onset (week)	IBD	Discontinuation of therapy	Drug-related event
**8**	Hepatic metastasis of a neuroendocrine tumour	4	UC	Yes	Possibly related
**53**	Bowel resection for inflammatory stenosis	24	CD	Yes, but for SLR	Not related
**89**	Hospitalization for acute flare	9	CD	Yes, but for SLR	Not related
**29**	*Staphylococcus aureus* sepsis	24	CD	Yes	Possibly related
**94**	Hospitalization for diarrhoea-associated hypopotassaemia	41	CD	No	Not related
**65**	Surgery for scoliosis	57	CD	No	Not related
**91**	Traumatic subarachnoid haemorrhage	12	UC	No	Not related
Patient no.	Non-serious adverse event	Point of onset (week)	IBD	Discontinuation of therapy	Drug-related event
**1**	Arthralgia	26	UC	No	Not related
**2**	Arthralgia	34	CD	No	Not related
**5**	Arthralgia	18	UC	No	Not related
**7**	Arthralgia	17	UC	No	Not related
**9**	Arthralgia	52	CD	No	Not related
**11**	Arthralgia	12	CD	No	Not related
**12**	Arthralgia	16	UC	No	Not related
**16**	Arthralgia	19	CD	No	Not related
**18**	Arthralgia	32	CD	No	Not related
**39**	Arthralgia	25	CD	No	Not related
**52**	Arthralgia	25	UC	No	Not related
**53**	Arthralgia	20	CD	No	Not related
**58**	Arthralgia	20	CD	No	Not related
**61**	Arthralgia	20	CD	No	Not related
**67**	Arthralgia	36	UC	No	Not related
**6**	Spondyloarthropathy	52	UC	No	Not related
**52**	Enthesitis	49	UC	No	Not related
**94**	Gout	37	CD	No	Not related
**6**	Cephalalgia	13	UC	No	Not related
**2**	Axillary abscess	14	CD	No	Possibly related
**37**	Respiratory infection	57	CD	No	Possibly related
**39**	Respiratory infection	51	CD	No	Possibly related
**35**	Thrush oesophagitis	24	CD	No	Possibly related
**60**	Erysipelas	27	CD	Yes, but for SLR	Possibly related
**63**	Herpes zoster	32	UC	No	Possibly related
**66**	Acute tonsillitis	25	UC	No	Possibly related
**69**	Otitis media	57	CD	No	Possibly related
**67**	Mastitis	20	UC	No	Possibly related
**2**	Uveitis	49	CD	No	Not related
**11**	Episcleritis	47	CD	No	Not related
**21**	Conjunctivitis	35	CD	No	Not related
**35**	Conjunctivitis	30	CD	No	Not related
**50**	Conjunctivitis	60	CD	No	Not related
**92**	Conjunctivitis	14	CD	No	Not related
**35**	Scleritis	36	CD	No	Not related
**39**	Adverse skin reaction	33	CD	No	Certainly related
**29**	Adverse skin reaction	16	CD	No	Certainly related
**44**	Adverse skin reaction	8	UC	No	Certainly related
**67**	Adverse skin reaction	56	UC	No	Certainly related
**69**	Adverse skin reaction	8	CD	No	Certainly related
**75**	Adverse skin reaction	5	CD	No	Certainly related
**22**	Erythema	18	CD	No	Possibly related
**57**	Perioral dermatitis	25	CD	No	Possibly related
**73**	Anaphylactic infusion reaction	40	CD	Yes	Certainly related
**76**	Anaphylactic infusion reaction	0	UC	Yes	Certainly related
**15**	Anaphylactic infusion reaction	0	UC	No	Certainly related
**51**	Hepatitis of unknown origin	55	CD	No	Possibly related
**51**	Gilbert’s syndrome	50	CD	No	Not related
**62**	Perianal abscess	17	CD	No	Not related
**62**	Perianal fistula	31	CD	No	Not related
**4**	IgA nephropathy	48	CD	No	Possibly related
**22**	Nephrolithiasis	35	CD	No	Not related
**48**	Nephrolithiasis	54	CD	No	Not related
**14**	Multinode struma	16	UC	No	Not related
**32**	Tinnitus	19	CD	Yes, but for SLR	Not related
**45**	Missed abortion	11	CD	No	Not related
**64**	Gastritis	12	CD	No	Not related
**67**	Reflux	48	CD	No	Not related
**91**	Anaemia	12	UC	No	Not related

CD, Crohn’s disease; IBD, inflammatory bowel disease; SLR, secondary loss of response; UC, ulcerative colitis.

The majority of AEs was not considered to be therapy-associated (42/66), which is highlighted by the fact that only in 4 patients the treatment was discontinued for potentially related safety issues. The incidence rate for AEs and SAE was 70.2 per 100 patient years (62.8 for AE; 7.4 for SAE) but considerably lower for therapy-associated AEs (23.4 per 100 patient years) and SAE (2.1 per 100 patient years), respectively. No safety concerns occurred during the observation period.

## Discussion

This long-term, prospective, non-interventional, longitudinal, observational cohort study aimed to investigate IBD patients with multiple infliximab switches, including reverse switching, in a real-world setting. In our cohort, there was a similar proportion of patients in clinical remission at baseline (time of the reverse switch) and after 48 weeks (80% vs 82%). The majority of patients (79%) received unchanged or lower dosing in the same or a prolonged infusion interval (89%) during the follow-up period. There was no signal regarding enhanced immunogenicity, with stable TL (median of 7.2 µg/ml at baseline vs 5.5 µg/ml at week 48). At baseline, 83% of patients were within or above the considered therapeutic TL range and 76% at week 48. Regression analysis verified no statistically significant changes in the TL (*p* = 0.26 in CD, *p* = 0.41 in UC) and 8-week-dosing (*p* = 0.87 in CD, *p* = 0.54 in UC) after the reverse switch. The median CRP levels remained below the threshold of 5 mg/l, which was considered to be normal. There was no statistically significant change in CRP levels after the reverse switch for CD (*p* = 0.30) and UC (*p* = 0.97) patients. These findings are in line with the data of another study, where 65.7%–76.9% of patients were in clinical remission 12 months after the initial switch, with no significant differences in CRP levels between the groups and no signs of need for dose intensification.^27^ Regarding clinical disease activity a multicentre, randomized, double-blind clinical trial comparing CT-P13 and originator infliximab in active CD, randomized patients to two groups who received induction and maintenance therapy till week 30 with CT-P13 and two groups with originator infliximab. At week 30, one of the CT-P13 groups was then maintained on CT-P13, and one was switched to originator infliximab. Similarly, one originator-infliximab group was maintained on infliximab, and the other was switched to CT-P13. At week 6, CT-P13 was found to be non-inferior to infliximab in the primary outcome of clinical response (69.4% vs 74.3%). Similar maintenance in efficacy was demonstrated in both groups at subsequent endpoints to 54 weeks.^28^ The respective cohort study from the Netherlands investigated the switch of 176 IBD patients (71% CD, 27.8% UC, 1.2% IBD unclassified).^27^ The cohort was divided into three groups: group 1 underwent a switch from originator infliximab on CT-P13 and subsequently on SB2 (39%), group 2 underwent a switch from originator infliximab on CT-P13 (45%) and group 3 a switch from CT-P13 to SB2 (15%). The primary outcome measure was clinical disease activity 12 months after the most recent switch. Remission was determined by physician’s choice and absence of concomitant steroid therapy. Secondary endpoints included faecal Calprotectin (fCP), CRP, discontinuation of the treatment and safety. There was no statistically significant difference in clinical disease activity 12 months after the index switch with 76.9% (*n* = 52), 76.9% (*n* = 26) and 65.7% (*n* = 70) of patients in clinical remission in group 1, 2 and 3. CRP- and fCP levels showed no significant differences between the three groups and no unexpected safety issues appeared. The authors concluded multiple switches to be effective and safe.^27^ Data regarding immunogenicity patterns were not released. A recently published study investigated 297 IBD patients (196 CD) for 7.5 months after switching on an infliximab biosimilar, including 205 patients with multiple switches. The study found a 9.4% discontinuation rate during the follow-up period and no significant differences between the groups (divided by the amount of switches) regarding clinical (*p* = 0.77), biochemical (CRP, *p* = 0.75) and faecal biomarker (fCP, *p* = 0.63) remission.^29^ Another study confirmed that double switching from the originator infliximab to CT-P13 and then to biosimilar SB2 did not impair the effectiveness, immunogenicity or safety of anti-TNF therapy after 54 weeks of follow-up.^30^ A different study reported outcomes with infliximab biosimilar to biosimilar switch in 271 IBD patients, including patients who were switched from infliximab originator to CT-P13 to SB2. Patients who underwent a double switch experienced no differences in efficacy or safety compared with those who had only one medication switch from infliximab reference product to SB2.^31^

Immunosuppressive co-medication was scarce in our cohort with 7.4% at baseline and an additional 6.3% at any time point during the follow-up. Despite the low rates of immunosuppressive co-medication, the vast majority of patients (97.7%) did not develop new ADA during the follow-up period and 3.4% lost initial ADA-positivity, showing the absence of general need for therapy-intensification after the reverse switch. However, patients in this cohort were introduced on infliximab with a median of nearly 3 years before the initial switch. There might be a substantial proportion of patients who initially received immunosuppressive co-medication and continued infliximab mono-therapy after reaching stable remission, following the top-down approach.

Regarding safety, an incidence rate of 62.8 per 100 patient years for AE and 7.4 per 100 patient years for SAE was documented, which is numerically higher than in our previous publication (17.8 AEs/100 patient years; 5.9 SAEs/100 patient years), but the majority of (S)AEs (42/66) was not considered to be related to infliximab therapy.^17^ Only four patients discontinued treatment due to safety issues (two anaphylactic reactions, one sepsis, one new hepatic metastasis in pre-existing neuroendocrine tumour). No unexpected safety issues appeared. Presumably because this cohort contains patients with long-term responses to infliximab treatment, the rates of suspected SLR remained low (5.7% of treatment discontinuations). Another reason for the low SLR might be the adequately high dosing of infliximab (8-week adjusted dosing 10.3 mg/kg in CD and 8.3 mg/kg in UC). Overall, 14.7% of patients discontinued therapy, which is in line with our previous findings after the first switch (29.2% discontinuation rate during 18 months) as well as the findings of a published study and within the expected range of annual risk for loss of response to infliximab of 13%.^17,32^ In another study, infliximab discontinuation rates between patients who reverse switched and patients who remained on biosimilar was under 10% after 1-year follow-up.^21^ Survival on treatment was significantly higher in patients with clinical remission at the time of inclusion with 93.0% (95% CI 0.87–0.99) versus 71.4% (95% CI 0.55–0.94) in non-remitters at week 48 (*p* = 0.005). These data might lead to the conclusion, that a reverse switch is not associated with increased loss of response and discontinuation rates in patients, especially if in stable remission.

There are currently only a very limited number of studies available, where non-medical reverse switching was performed from an anti-TNF biosimilar to the originator in patients with IBD. In a recently published prospective observational study 174 patients with IBD (136 with CD and 38 with UC) were switched from the biosimilar CT-P13 to originator infliximab due to reimbursement policies.^19^ Here, only 8% of patients had been previously exposed to the originator infliximab. There was no significant difference in the proportion of patients in clinical remission before the switch (82.5% with CD and 82.9% with UC) and at week 24 (CD 76.3% with CD and 84.9% with UC). Additionally, no significant changes were observed in TLs, or ADAs after the reverse switching procedure.^19^ In another study, 75 of 758 patients (9.9%) were reverse switched from CT-P13 to infliximab originator because of gastrointestinal symptoms or dermatological adverse effects. Here, drug persistence was equal between patients maintained on CT-P13 and patients who were reverse switched in multivariable analyses. No clinically relevant differences were detected between the pharmacokinetics of CT-P13 and originator infliximab.^21^

Limitations of our study are the limited sample size, lack of objective clinical activity data regarding fCP levels or endoscopic activity. There was also no control group of patients continuing infliximab biosimilar SB2, as all patients were transitioned to originator infliximab in the reverse switching procedure.

Additionally, there is a selection bias in this study, we want to address, as only patients were included with a long time-response to infliximab before the reverse switch, which is best reflected by the median infliximab treatment duration of 33 months in our patient cohort before reverse switching took place. This highlights the importance of the timing of switching, which might potentially be more favourable in patients who have stable disease with steady-state infliximab levels under long-standing infliximab treatment in comparison to the first year of therapy, where the loss of response rates might be higher with less favourable outcomes of switching procedures. Nevertheless, the central question, if multiple infliximab switches have an influence on the clinical outcome of IBD patients, is not affected by the selection bias and is comparable to other studies. Strengths of our study are the close patient follow-ups with a high density of data and a long observational period with a median of 158 weeks (range 105–170) and the consideration of not only clinical disease activity but also safety, biochemical (CRP) activity and the pharmacokinetics and immunogenicity pattern (TL, ADA). A further advantage of the cohort is that all patients have had previous exposure to the originator infliximab before being treated with the biosimilar SB2 and the reverse switching procedure to originator infliximab (multiple switches).

We want to conclude, that multiple switches between originator infliximab and the biosimilar SB2 have no clinically meaningful impact on the efficiency of therapy, immunogenicity pattern or safety in this long-standing IBD cohort. Therefore, multiple switches, including reverse switching between infliximab and its biosimilars may be feasible. However, there might be other challenges in switching to other anti-TNF agents. While multiple switches of intravenous infliximab applications are technically feasible, switching between adalimumab biosimilars also includes needed adjustments to the varying autoinjector devices used by the different adalimumab biosimilar companies. This can be a significant challenge for nurses and patients, as these devices differ in needed operation steps, needle size, grip surface, shape, visual control and trigger button among others, which all can decrease tolerance and adherence, potentially resulting in treatment failure. The process of non-medical switching was implemented to keep treatment costs low for the healthcare payers. Non-medical switching is mostly the result of federal or hospital demands and it should ideally be ensured that the financial gain is not allocated to the healthcare payer in general, but should rather be shifted to improve patient care, which could be done by expanding the number of nurses or physicians taking care of the IBD patients. It also needs to be mentioned that one potential reason for drug discontinuation, the nocebo effect, has been recognized as an important clinical concern in the current era of biosimilars since it has a detrimental influence on drug adherence, patient quality of life and treatment outcomes.^33^ A recent study demonstrated a temporary yet discernible nocebo effect in the first 16 weeks following non-medical switching that was not sustained at week 32 in IBD patients. Thus, a patient approach with close clinical follow-up and disease monitoring is warranted for overcoming these negative patient perceptions.^34^

## Conclusion

Our data may encourage clinicians to switch infliximab compounds in IBD patients due to non-medical reasons, though our data are restricted to two switching procedures and therefore the observed findings must not be generalized to encourage clinicians to repeatedly switch between all infliximab biosimilars (interchangeability) available, as more data need to be generated in this regard. Ideally, the time point of switching would be at the time of clinical remission. Nevertheless, larger, well-powered studies with matched controls, robust capture of pharmacovigilance data with long-term follow-ups and multiple switching sequences are necessary before multiple switches between infliximab formulations can become an evidence-based recommendation.^35^

## Supplemental Material

sj-pdf-1-tag-10.1177_17562848241301887 – Supplemental material for Reverse switching from the biosimilar SB2 to the originator infliximab in previously switched patients with inflammatory bowel diseases: results of a prospective long-term cohort study Supplemental material, sj-pdf-1-tag-10.1177_17562848241301887 for Reverse switching from the biosimilar SB2 to the originator infliximab in previously switched patients with inflammatory bowel diseases: results of a prospective long-term cohort study by Sarah Fischer, Moritz Donhauser, Sarah Cohnen, Konstantin Fietkau, Marcel Vetter, Maria Grübel-Liehr, Peter Dietrich, Timo Rath, Angelika Wilfer, Ludmilla Sologub, Sabine Krebs, Frank Dörje, Daniel Nagore, Sebastian Meyer, Markus F. Neurath and Raja Atreya in Therapeutic Advances in Gastroenterology

## Declarations

**Ethics approval and consent to participate:** Patients gave written informed consent regarding blood sampling before participating in the study. The sample collection was previously approved by the ethical committee and the institutional review board of the Friedrich-Alexander-Universität of Erlangen-Nürnberg (proposition approval number: 426_20B).

**Consent for publication:** The participating patients gave consent to publish the analysed data in an anonymized form.

**Availability of data and materials:** Individual participant data collected during the study after de-identification are available for the present study. Requests are reviewed and approved by the corresponding author based on scientific merit. All data provided are anonymized to respect the privacy of patients who have participated in the trial, in line with applicable laws and regulations. Proposals should be directed to corresponding author at raja.atreya@uk-erlangen.de; to gain access.
